# Achy Breaky Heart: A Rare Case of Myopericarditis Secondary to Mesalamine in a Patient With Inflammatory Bowel Disease

**DOI:** 10.7759/cureus.52587

**Published:** 2024-01-19

**Authors:** Nikhila Appala, Hima Veeramachaneni, Anshika Khare, Preeyanka Sundar

**Affiliations:** 1 Internal Medicine, Kasturba Medical College, Manipal, IND; 2 Gastroenterology and Hepatology, Emory University School of Medicine, Atlanta, USA

**Keywords:** life threatening complication, drug-induced adverse reaction, ibd, mesalamine, myopericarditis

## Abstract

Mesalamine is a first-line drug used in the treatment of inflammatory bowel disease (IBD), specifically ulcerative colitis (UC), with side effects ranging from gastrointestinal effects to cardiotoxicity. We present a rare case of mesalamine-induced myopericarditis in a patient with IBD, who presented with epigastric pain and was found to have elevated an c-reactive protein (CRP) in the absence of chest pain and any other gastrointestinal symptoms. This case highlights the importance of including myopericarditis as a differential for IBD patients on mesalamine with an isolated elevated CRP, especially within the first month of initiating this medication, as drug cessation usually leads to immediate clinical improvement.

## Introduction

Among anti-inflammatory drugs used in the treatment of inflammatory bowel disease (IBD), mesalamine is a common one, especially for ulcerative colitis (UC) [1-3]. Pericarditis can present as an extraintestinal presentation of IBD, 0.23% in UC patients and 0.19% in Crohn’s disease, as well as an adverse effect of the treatment [4,5]. Myopericarditis is a rare but lethal complication in those who take this drug [6,7]. Recognizing this rare and life-threatening complication is crucial in diagnosing and treating myopericarditis and ultimately preventing severe detrimental cardiovascular effects and death [3,5].

## Case presentation

A 48-year-old male with a history of UC on treatment with Ustekinumab was admitted from the clinic for constant, progressively worsening, non-radiating, epigastric pain for two weeks with an elevated c-reactive protein (CRP). He had no changes in bowel movements, recent viral illness, no cardiac history, or family history of coronary artery disease. His last colonoscopy was three months prior, which showed mildly active colitis, and he was treated with prednisone and the addition of mesalamine to Ustekinumab with an improvement in symptoms. The patient restarted the mesalamine again two weeks before admission when the patient described epigastric pain, and there was concern for an ongoing IBD flare with the outpatient physician.

**Figure 1:**
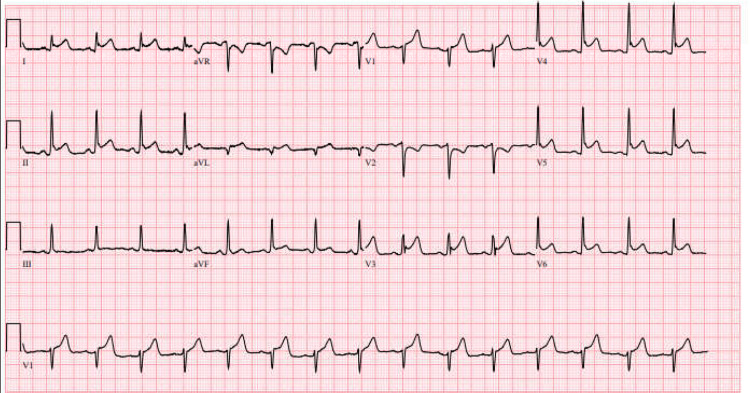
Electrocardiogram showing diffuse ST elevation.

**Figure 2:**
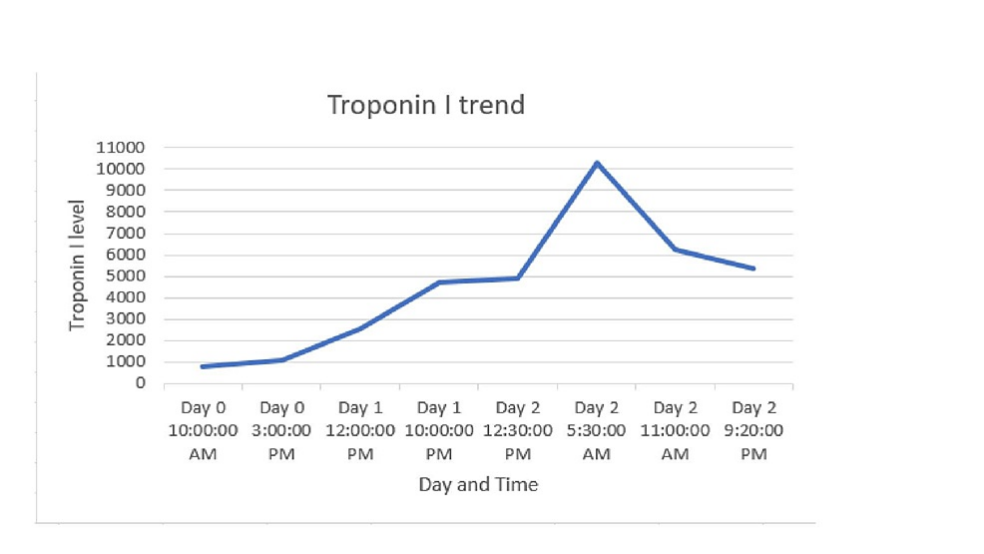
Troponin trend graph over the course of the hospitalization

Upon admission, vitals were remarkable for tachycardia in the 130s, and labs revealed a normal hemoglobin level of 14, an elevated CRP of 129, and a high sensitivity troponin-I level of 1096. The patient had no symptoms of chest pain or breathlessness, and CT scan of the chest, abdomen, and pelvis were normal. The tachycardia persisted after fluid boluses, GI cocktail, and acetaminophen, but abdominal discomfort improved. Due to the elevated CRP and epigastric pain with IBD, the plan was to pursue esophagogastroduodenoscopy and flexible sigmoidoscopy to assess for peptic ulcer disease or active colitis. However, a repeat high-sensitivity troponin revealed a rise to 2,515 and a peak at 10,000 (Figure 1, Table 1), with diffuse ST elevation seen on the electrocardiogram (Figure 2). An echo performed demonstrated that everything was normal except a low normal systolic function (EF of 50-55%). Cardiology was consulted, and he was taken urgently for a coronary angiography, which did not reveal any ischemic cardiac disease. A diagnosis of mesalamine-induced acute myopericarditis was made, and he was managed with cessation of the drug, oral steroids, and colchicine. He had an improvement in his epigastric pain and was discharged two days later with tapered steroids and high-dose ibuprofen.

**Table 1:** Lab trends over the course of the hospitalization ESR: erythrocyte sedimentation rate; CRP: c-reactive protein

		ESR (reference range: 1-15 mm/hr)	CRP (reference range: <= 10.0 mg/L)	Troponin-I (Reference range: 0- 004ng/mL)
11/22/2023	10:25	-	-	785
11/22/2023	15:00	-	-	1096
11/23/2022	1:25	27	129.7	2515
11/23/2022	21:55	60	228.4	4729
11/24/2022	12:30	-	-	4892
11/24/2022	5:30	-	-	10289
11/24/2022	11:00	-	-	6242
11/24/2022	21:20	-	129.6	5347
12/02/2022	9:53	-	2.2	-

## Discussion

Many proposed theories about mesalamine-induced pericarditis include direct cytotoxic damage and humoral or cell-mediated mechanisms [3]. The most common theory is a humoral-mediated hypersensitivity reaction, in which antibodies against mesalamine cross-react with cardiac tissue leading to inflammation [6,8-10].

In this case, the diagnosis of myopericarditis was made clinically based on the constellation of symptoms and laboratory findings in correlation with the timing of the recent initiation of mesalamine therapy. In contrast, pericarditis developing as an extraintestinal manifestation occurs independent of the IBD and may be the initial presentation [5,11-12].

Myopericarditis classically presents as fever, chest pain, and dyspnea within four weeks after starting the drug, with a physical exam revealing tachycardia and pericardial rub in some patients [2,13]. EKG shows nonspecific ST segment changes or T wave changes and, as in this case, can show concave ST elevation and PR depression in precordial leads, which is classically seen with pericardial inflammation [3,14]. Laboratory investigations will have elevated erythrocyte sedimentation rate (ESR), CRP, cardiac troponin, and N-terminal pro-B-type natriuretic peptide (NT-proBNP). Cardiac imaging may show LV systolic dysfunction with pericardial effusion with or without tamponade effect [3,13].

## Conclusions

Myopericarditis, based on etiology, is treated by targeting the underlying causes and, as in this case, stopping the offending agent and ensuring not to use it in the future. Several cases in the literature have shown that symptoms resolve within one week of discontinuing the drug and starting steroids. In other cases, where there is no identifiable cause or is due to an untreatable cause such as viruses, supportive management is administered to avoid complications such as arrhythmias and heart failure. It is also important to note that patients who develop hypersensitivity to mesalamine will most likely not tolerate sulfasalazine as well.

Complications of myopericarditis include tachyarrhythmias, heart block, left ventricular dysfunction, dilated cardiomyopathy, and cardiac arrest. Gastroenterologists need to keep myopericarditis in the differential when patients on mesalamine present with this constellation of signs and symptoms.
